# Aflatoxin Exposure and Human Health with a Focus on Northern Latin America

**DOI:** 10.3390/toxins18010058

**Published:** 2026-01-22

**Authors:** Karen A. Corleto, Christian S. Alvarez, Manuel Ramirez-Zea, John D. Groopman, Katherine A. McGlynn

**Affiliations:** 1 Division of Cancer Prevention, National Cancer Institute, Rockville, MD 20850, USA; karen.corleto@nih.gov; 2 Division of Cancer Epidemiology and Genetics, National Cancer Institute, Rockville, MD 20850, USA; 3 Division of Intramural Research, National Institute on Minority Health and Health Disparities, Rockville, MD 20852, USA; christian.alvarez@nih.gov; 4 Center for the Prevention of Chronic Diseases, Institute of Nutrition of Central America and Panama, Guatemala City 1188, Guatemala; mramirez@incap.int; 5 Departments of Environmental Health and Engineering and Epidemiology, Bloomberg School of Public Health, Johns Hopkins University, Baltimore, MD 21205, USA; jgroopm1@jhu.edu

**Keywords:** aflatoxin, maize, liver cancer, Latin America

## Abstract

Aflatoxins, mycotoxins produced by *Aspergillus flavus* and *Aspergillus parasiticus*, were discovered sixty-five years ago and remain a significant public health threat, particularly amid increasing instances of extreme weather events. Of the four principal forms of aflatoxins found in foods (B_1_, B_2_, G_1_, and G_2_), aflatoxin B_1_ is the most potent carcinogen. Aflatoxins commonly contaminate a variety of foodstuffs, with maize being among the most susceptible. Chronic exposure to aflatoxins has been linked to liver cancer, childhood stunting, gallbladder cancer, and other adverse health effects. Due to public health concerns related to the consumption of aflatoxin-contaminated foods, most countries have established regulatory limits. Here, we present estimated aflatoxin exposure per day derived from human biomarker data across many studies around the world spanning more than forty years. We specifically focus on the impact of dietary aflatoxin in northern Latin America, where assessment of the total problem remains limited. These findings suggest a multipronged toolkit could mitigate aflatoxin exposure in the region, which would help to decrease the health burden.

## 1. Introduction

Aflatoxins, identified as disease-causing mycotoxins sixty-five years ago [1,2], continue to be significant public health issues due to their toxic, mutagenic, and carcinogenic properties [3]. Primarily produced by the fungi *Aspergillus flavus* and *Aspergillus parasiticus* [4,5], approximately twenty aflatoxins have been described [2], but the four principal forms that dominate human exposure are aflatoxin B_1_ (AFB_1_), B_2_ (AFB_2_), G_1_ (AFG_1_), and G_2_ (AFG_2_) [4]. Of these forms, AFB_1_ is the major aflatoxin driving health concerns [6].

Although other routes of exposure occur, human exposure to AFB_1_ occurs predominantly through the consumption of contaminated foods. Various foodstuffs have tested positive for aflatoxin contamination, including spices, pulses, beans, tea, cocoa, milk, grains, and groundnuts [3,6,7]. The major sources of aflatoxin in the human diet, however, are maize, groundnuts and other grains [4,6,8]. AFB_1_ contamination can be challenging to combat as it can develop both pre- and post-harvest, with weather conditions including rainfall, temperature, and humidity converging to produce environmental conditions optimal for mold growth [6,8].

Due to widespread environmental contamination by aflatoxins and the negative health implications for both humans and other animal species, a number of countries have established regulatory limits on aflatoxins in foodstuffs, but there is no global consensus on regulatory standards.

## 2. Regulatory Limits on Aflatoxins

Regulatory limits for aflatoxins vary widely by country and food item. For example, the European Union (EU) regulations are some of the most stringent, with limits for both total aflatoxins (4–15 parts per billion, ppb), and for AFB_1_ (2–12 ppb) [9]. The EU regulations vary by commodity but are most strict for ready-to-eat foods. In contrast, the United States has only one limit (20 ppb) for all food items [10], while Mexico has a limit of 20 ppb for maize grain but a lower level (12 ppb) for nixtamalized maize products [11,12]. Guatemala also has a limit of 20 ppb (total aflatoxins) for maize and maize products [12,13].

Despite the existence of regulatory limits, aflatoxin contamination remains a concern in many countries. A risk assessment that evaluated the effectiveness of aflatoxin regulatory standards in protecting against liver cancer concluded that most standards limited lifetime risk to below 1 case per 10,000 individuals [14]. However, the report also found that in some parts of Latin America, regulatory standards did not meet this level of protection [14]. In addition, regulatory standards are only effective if they are enforced and many countries lack the capacity to do so.

Table 1 shows the estimated intake of total aflatoxins depending on the regulatory standard and the grams of a dietary item consumed. To calculate daily aflatoxin exposure, 3% of the intake of contaminated food was used to estimate AFB_1_ µg/day. The calculation assumes a steady state of adduct accumulation over 30 days, as previous work indicates that 2.9% of the daily dietary exposure is converted to the aflatoxin albumin adduct.

## 3. Evaluating Aflatoxin Exposure

While global exposure to aflatoxins from contaminated foods is well documented, estimating the putative dose to humans has been a goal of aflatoxin biomarker research. Knowledge of the metabolism and formation of specific covalent aflatoxin adducts in DNA and proteins has been deployed in many epidemiologic studies. In recent years the recognition that the aflatoxin-lysine adduct formed in albumin is an integrative biomarker of exposure has been of great significance in the field [15]. To put the AFB_1_ levels in northern Latin America in context, Table 2 summarizes studies of Groopman and colleagues of AFB_1_-lys adduct levels and converts those levels to estimated daily aflatoxin exposure in µg, using the formula mentioned above [16,17].

**Figure 1:**
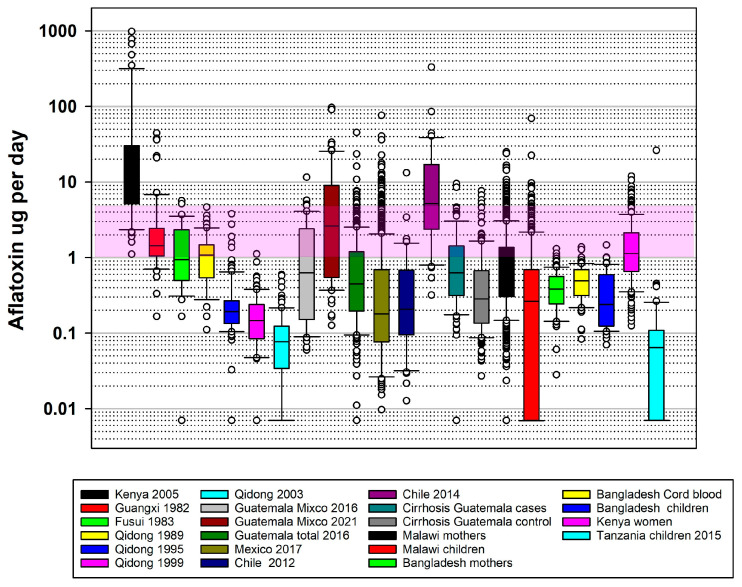
Graphical representation of daily aflatoxin exposure in µg for studies in Kenya (2005) [25,26]; Guangxi, Fusui, and Qidong [17,21]; Guatemala [23,24]; Mexico [29,30]; Chile [36,37]; cirrhosis in Guatemala [22]; Malawi [18]; Bangladesh [18,19,20]; Kenyan women [27,28]; and Tanzanian children [31,32]. The area highlighted represents the 1 μg to 5 μg per day range where many human health effects have been documented. The open circles represent individual data points that are either below the 5th percentile or above the 95th percentile.

As seen in Table 2**,** the lowest mean daily aflatoxin exposure (0.01 ± 0.02 µg/day) seen in these studies was in 2012 in Qidong, China. This low level was achieved after the implementation in Qidong of the switch in dietary staple from maize to rice. In comparison, the highest exposure (91.1 ± 201.42 µg/day) in Table 2 occurred during an outbreak of acute aflatoxicosis in Kenya in 2004 [33,34,35]. Figure 1 displays the results of many of these studies plotted side by side.

## 4. Maize Consumption as a Vehicle for Aflatoxin Exposure

In northern Latin America, maize has been a dietary staple for millenia. Numerous foods, such as tortillas, tamales, gorditas, and pupusas, are made from nixtamalized maize flour, which is maize grain that has been cooked in a solution of calcium hydroxide prior to being ground [38,39,40]. Environmental conditions (temperature, humidity, and rainfall) in the region can facilitate pre- and post-harvest AFB contamination [3]. Extreme weather events, including droughts and unseasonal rains, further increase the risk of AFB contamination, and rising temperatures and humidity are projected to enhance pre-harvest aflatoxin contamination of susceptible crops [8,41].

Of the food items made from maize, tortillas are consumed in the greatest quantity. For example, in Veracruz City, Mexico, reports indicate that tortilla intake averages 118.4 g (person/day) [42]. In a recent report from Mexico that evaluated maize consumption using the country’s National Health and Nutrition Survey (ENSANUT), the median intake of maize was 307 g/d, mainly attributed to tortilla intake (77%) [29]. The high consumption of tortillas is of particular concern as tortillas have been demonstrated by several studies to be contaminated with AFB. In the Mexican state of San Luis Potosí, an evaluation of tortillas revealed that 18% of the samples exceeded Mexico’s limit (12 ppb) for AFB_1_ [43], while in Mexico City, 4.5% of nixtamalized maize samples were reported to have detectable levels of aflatoxins [39].

In Guatemala, a cross-sectional evaluation of 461 adults found median AFB_1_ levels of 7.5 pg/mg albumin among women and 11.4 pg/mg albumin among men [44]. For reference, a level of 17.5 pg/mg albumin is an adduct burden arising from a daily exposure to 1 µg AFB_1_ per day for 30 days [17]. In the cross-sectional study, the median AFB_1_ levels were higher among individuals in rural areas (14.0 pg/mg albumin) than in urban areas (4.9 pg/mg) [44]. The study also found a significant relationship between tortilla consumption and levels of AFB_1_-albumin adducts [45]. A separate study of Guatemalan women who worked as tortilla makers reported an average weekly consumption of maize to be 3470.5 g, primarily in the form of tortillas. Tortilla sampling found that 11% had detectable levels AFB_1_ [46]. In a study from Honduras, 65% of maize and maize products were contaminated with aflatoxin, with the mean total aflatoxins ranging from 1.06 µg/kg to 6.23 µg/kg [47].

A 2014 examination of global maize consumption estimated that the highest levels of consumption in the western hemisphere were in Mexico (267 g/person/day), Guatemala (187 g/person/day), and Honduras (169 g/person/day) [48]. Thus, with the aflatoxin regulatory limit in Mexico of 20 ppb (20 µg per kg), the consumption of 267 g/person/day in Mexico would result in an aflatoxin exposure of roughly 3.2 µg/day. A similar calculation for Guatemala results in an aflatoxin exposure of roughly 3.7 µg/day. In addition, localized reports from many areas have found that maize products are being consumed at a level of more than 260 g/day [29,45,46,47]. These data indicate that contamination levels in several regions exceed the regulatory limits, making these regulations insufficient/ineffective to protect human health.

## 5. Health Burden of Aflatoxin Exposure in Latin America

Aflatoxin has been associated with a number of health-related conditions, with liver cancer, after acute aflatoxicosis, being one of the most fatal. Liver cancer is the sixth most commonly diagnosed cancer worldwide and the third largest contributor to cancer mortality [49]. The relationship between aflatoxin exposure and liver cancer is well documented, leading the International Agency for Research on Cancer to classify aflatoxin as a group 1 human carcinogen [5,17,50,51,52,53]. In Latin America, while there are few established cancer registries, it is estimated that Guatemala has the highest age-standardized liver cancer incidence rate (15.5 per 100,000 persons) in the western hemisphere [24]. In contrast, the age-standardinzed liver cancer incidence rate in the U.S. is 6.8/100,000 persons

Aflatoxin is not only a risk factor itself but can be an even more potent risk factor when it co-occurs with other factors, including alcohol, obesity, and type II diabetes, as well as chronic infections with hepatitis C virus and hepatitis B virus (HBV) [24,54,55,56,57]. The synergism between aflatoxin and HBV was well demonstarted in a study conducted in Shanghai, China, that found the risk of liver cancer was 3.4-fold higher among persons exposed to AFB_1_, 7-fold higher among persons who were HBV(+), and 59-fold higher among persons with both factors [58]. This synergistic relationship was also subsequently observed in other cohorts [51]. In Latin America, HBV prevalence estimates tend to be lower than in other areas of the world, although comprehensive prevalence data for the region are very limited [59]. For example, a study conducted in Guatemala that examined both aflatoxin and HBV found that the prevalence of aflatoxin was high but the prevalence of HBV was low [22,23,24].

Although HBV may not play a significant role in liver cancer in Latin America, factors such as obesity and type II diabetes [24,57] may also increase the risk of liver cancer among persons exposed to aflatoxin. In Latin America, it is estimated that 30.5% of adults live with obesity, and 64% are overweight [60]. With the increase in obesity rates, type II diabetes has also increased, with estimates that in the next 20 years there will be a 50% increase in type II diabetes in the region [60]. Obesity and type II diabetes also are risk factors for metabolic dysfunction-associated steatotic liver disease (MASLD), which itself is a risk factor for liver cancer [61]. In Guatemala, a study reported a significant association between AFB_1_-albumin adduct levels and diabetes [44]. Although the mechanism by which AFB_1_ increases risk of diabetes is uncertain, the relationship may be due to AFB_1_ causing inflammation and oxidative stress, impairing insulin secretion and disrupting carbohydrate metabolism. More research is needed to improve the understanding of the relationship between aflatoxins, metabolic disorders, and other factors in affecting liver cancer risk in Latin America.

In addition to liver cancer and metabolic disorders, aflatoxin has been associated with childhood stunting, both independently and in conjunction with other risk factors [4]. Hypotheses on the mechanism by which aflatoxin influences stunting include impairment of the immune system, effects on the IGF-1 axis, and an effect on environmental enteric dysfunction (EED) [4,62]. Among Latin American countries, Guatemala has a notably high level of stunting, with a prevalence ≥ 40% [62], and aflatoxin exposure has been negatively correlated with child height-for-age-z-scores [63]. A cross-sectional study conducted in low-income African countries found that children with both stunting and underweight had the highest levels of aflatoxin albumin adducts as measured by enzyme-linked immunosorbent assay (ELISA) (132.27 pg/mg albumin), followed by children with stunting alone (109.41 pg/mg albumin), followed by children without stunting (75.01 pg/mg albumin) [64]. In addition, a study in Malawi that used mass spectrometry reported that aflatoxin exposure was negatively associated with child growth [65]. Collectively, a published paired study can be used to interconvert data from ELISA with mass spectrometry-derived aflatoxin albumin adducts [25].

Other health-related conditions have also been linked to chronic dietary exposure to aflatoxins. It has been suggested that aflatoxins may influence innate and adaptive immunity [54,66], with some human studies providing evidence that aflatoxin can have immunosupressive effects in children and adults [67,68]. Gambian children with higher exposure to aflatoxin had reduced levels of the antibody secretory IgA in their saliva [67]. In adults from Ghana it was found that aflatoxin exposure influences cellular immune status [68]. Further research, particularly in human populations, is required to better understand how aflatoxin exposure affects immunity. Perhaps the results of studies of aflatoxin exposure and oncogenic human papillomaviruses (HPV) are related to effects on the immune system. Studies in both Kenya and Mexico have indicated an association between aflatoxin and oncogenic HPV in women [27,28,69,70]. Aflatoxin exposure may also be related to the development of gallbladder cancer, as reported by studies conducted in Chile and Shanghai [36,37,71,72]. There are also investigations into how aflatoxin-induced oxidative stress and reductions in nuerotransmitter levels may impact neurogenerative disease [54,73].

## 6. Toolkit of Interventions to Tackle Aflatoxin Exposure in Latin America

The regulation of aflatoxins in food and the strict enforcement of those regulations are critical components of a primary prevention approach to aflatoxin control. In conjunction with regulatory standards, aflatoxin urinary and blood biomarkers should be used to monitor exposure, providing a metric of the efficacy of the regulatory standards. Urinary biomarkers (AFB_1_, AFM_1_, and AFB_1_-N^7^-Gua DNA-adduct) are suitable for measuring short-term exposure [58,74], while serum AFB_1_-lys adduct levels are the best estimates of longer-term exposure. In humans, the average lifetime for albumin is approximately thirty days [75]. Therefore, albumin adduct formation provides a more comprehensive picture of aflatoxin exposure that estimates average aflatoxin exposure per day over a 30-day period. Using AFB_1_ metabolites to estimate daily aflatoxin exposure in Latin America would be an effective tool to monitor acute exposure or the average daily exposure over a longer time at the individual or population level. Effect monitoring programs could help governments gauge the effectiveness of regulatory measures and aid in making informed decisions about aflatoxin control. To most effectively conduct a monitoring program, there is a demonstrable need for capacity building and infrastructure development to establish aflatoxin monitoring laboratories throughout Latin America.

Due to the importance of preventing crop contamination from aflatoxins, pre-harvest contamination prediction models have been developed [76,77]. The most recent models use a meteorology-driven epidemiological approach, such as the model described by Stutt and colleagues that encompasses the maize supply chain from planting to delivery [76]. Biological control is also a possible approach to pre-harvest aflatoxin management. Some of the pre-harvest techniques include competitive exclusion using non-aflatoxigenic strains and microbial bio-fungicides [2,3,78,79,80]. During postharvest, proper drying, including such strategies as controlled environment drying, sun drying on platforms, and removing damaged corn, can aid in the reduction of aflatoxin contamination [12]. Field drying of maize under humid conditions in Mexico and northern Central America can result in moisture levels (14–18%), which are higher than the recommended range to reduce aflatoxin contamination (12–14%) [12]. Adequate storage with low humidity and temperature conditions also helps to minimize fungal growth [12].

In addition to pre- and post-harvest strategies for aflatoxin reduction, cooking can also reduce the amount of aflatoxin in foods. Roasting at high temperatures has been shown to decrease aflatoxin levels 50–90% [81]. Boiling can also be an effective strategy, as boiling rice at 100 °C for 12 min has been reported to decrease aflatoxin levels [82]. In Mexico and Central America, nixtamalization of maize is a common practice, and most maize products consumed are nixtamalized [12,39,45,83]. While some studies have found that nixtamalization reduced aflatoxin by 88% (44.99 ppb to 5.26 ppb) [38], the process is not entirely successful as aflatoxin contamination continues to be an issue in Latin America.

While primary prevention is always the optimal strategy, secondary prevention strategies, such as chemoprevention to detoxify consumed aflatoxins, have been proposed. AFB_1_ is bioactivated into aflatoxin-8,9-epoxide (AFBO), the metabolite that reacts with DNA to form adducts. To interrupt adduct formation, phase II metabolism then plays an important role in detoxification. Glutathione S-transferases conjugate glutathione to AFBO to facilitate eventual excretion in urine [52,84]. Agents that promote the induction of glutathione S-transferases can therefore assist in the detoxification process. Two agents that have shown a positive effect on glutathione S-transferase induction are the pharmaceutical Oltipraz and a natural compound found in cruciferous vegetables, sulforaphane [85,86,87]. In a placebo-controlled trial, Oltipraz at a dose of 500 mg taken weekly for one month was demonstrated to decrease median urinary levels of the metabolite AFM_1_ by 51% [87,88]. In a study of sulforaphane, 2 weeks of broccoli sprout infusion resulted in modest decreases in urinary levels of aflatoxin N^7^-guanine [85]. In the study, there was high variability among individuals in sulforaphane levels, which correlated with decreases in aflatoxin N^7^-guanine [85]. Other bioactive compounds that have shown promising results are polyphenols from curcumin and green tea [84,89]. To our knowledge, these secondary chemoprevention strategies have not yet been tried in Latin America.

## 7. Conclusions

Aflatoxins threaten human health in a myriad of ways across the lifespan. The carcinogenic effects of AFB_1_ on the liver are of particular concern. In addition, the effects on childhood stunting, oncogenic HPV infection, and gallbladder cancer are of significant concern in areas where contamination is common. Due to fluctuating environmental conditions, this aflatoxin contamination could greatly increase. There is a strong need to implement effective aflatoxin monitoring systems, in areas such as Latin America, where staple dietary foods such as maize are commonly contaminated. Urinary and blood biomarkers produced during aflatoxin metabolism are useful for monitoring exposure in individuals and for measuring the effectiveness of mitigation strategies. The effectiveness of both pre-harvest and post-harvest techniques can be measured by biomonitoring a population’s daily aflatoxin exposure. The success of secondary prevention methods can also be determined. Overall, the use of aflatoxin biomarker monitoring in Latin America could guide regulatory efforts and assist in making informed decisions on intervention strategies.

## Figures and Tables

**Table 1:** Estimated daily aflatoxin exposure by regulatory limits and intake of contaminated foods.

Contaminated Food Consumption Level per Day (g)	Total Aflatoxins Regulatory Limits (ppb)	Estimated Aflatoxin Intake (ug)
25	4	0.10
	10	0.25
	15	0.38
	20	0.50
50	4	0.20
	10	0.50
	15	0.75
	20	1.00
100	4	0.40
	10	1.00
	15	1.50
	20	2.00
250	4	1.00
	10	2.50
	15	3.75
	20	5.00
350	4	1.40
	10	3.50
	15	5.25
	20	7.00

**Table 2:** Daily aflatoxin exposure (µg) for various studies.

Country	Year	Details	*n*	Aflatoxin (SD)	95% CI	Min.	Max.	%ND *	Ref.
Bangladesh	2008–2011	Preg. women 1st trimester	63	0.35 (0.22)	(0.29, 0.41)	ND	1.14	6.4	[18,19,20]
		Preg. women 3rd trimester	63	0.48 (0.25)	(0.42, 0.55)	ND	1.31	1.6	
		Cord blood	63	0.51 (0.27)	(0.44, 0.57)	0.08	1.38	0	
		Child 24 mos.	63	0.37 (0.30)	(0.30, 0.45)	ND	1.47	1.6	
China	1982	Guangxi	76	4.01 (8.30)	(2.11, 5.91)	0.17	44.38	0	[17,21]
	1983	Fusui	31	1.55 (1.40)	(1.04, 2.06)	ND	5.66	3.2	
	1989	Qidong	74	1.20 (0.88)	(1.00, 1.40)	0.11	4.66	0	
	1995	Qidong	100	0.33 (0.52)	(0.22, 0.43)	ND	3.79	2	
	1999	Qidong	100	0.20 (0.19)	(0.16, 0.23)	ND	1.12	8	
	2003	Qidong	100	0.10 (0.12)	(0.08, 0.13)	ND	0.6	24	
	2009	Qidong	100	0.06 (0.14)	(0.03, 0.09)	ND	0.81	77	
	2012	Qidong	100	0.01 (0.02)	(0.01, 0.02)	ND	0.16	92	
Guatemala	2015	Cirrhosis cases	99	1.18 (1.54)	(0.87, 1.49)	ND	9.52	1	[22]
		Controls	200	0.67 (1.11)	(0.52, 0.83)	0.03	7.62	0	
	2016	Escuintla	93	7.73 (12.82)	(5.09, 10.37)	ND	87.38	4.3	[23,24]
		Mixco	60	1.50 (2.06)	(0.97, 2.03)	0.06	11.57	0	
		Sololá	60	1.97 (3.13)	(1.16, 2.78)	0.18	23.51	0	
		Suchitepéquez	82	2.02 (2.43)	(1.49, 2.56)	0.09	16.12	0	
		Quiche	101	1.33 (4.93)	(0.36, 2.31)	ND	45.2	1	
Kenya	2005	Aflatoxicosis outbreak	55	91.1 (201.42)	(36.65, 145.55)	1.11	981.1	0	[25,26]
	2015–2016	Women baseline	120	1.93 (1.95)	(1.58, 2.29)	0.13	10.61	0	[27,28]
		Women 12-mo. follow-up	115	1.55 (1.62)	(1.25, 1.85)	0.14	11.93	0	
Malawi	2011–2015	Preg. women baseline	255	1.04 (1.43)	(0.87, 1.22)	ND	15.57	4.7	[18]
		Preg. women GA 36 wks.	255	1.20 (1.77)	(0.98, 1.41)	ND	10.73	4.7	
		Women 6 mos. postpartum	230	1.86 (3.31)	(1.43, 2.29)	0.04	25.04	0	
		Infant 6 mos. old	230	0.41 (1.00)	(0.28, 0.54)	ND	8.53	43.5	
		Infant 18 mos. old	231	1.49 (4.98)	(0.85, 2.14)	ND	69.28	8.7	
Mexico	2018–2019	South and eastern Mexico	855	1.03 (3.77)	(0.78, 1.28)	0.01	76.23	0	[29,30]
Tanzania	2018	Haydom (rural)	545	1.76 (3.00)	(1.51, 2.02)	ND	42.98	0.2	[31,32]

Aflatoxin is represented in µg/day; calculations were estimated from plasma serum AFB1-albumin adducts. Abbreviations: pregnant (Preg.), month (mo.), months (mos.), gestational age (GA), standard deviation (SD), confidence interval (CI), nondetectable (ND), minimum (Min.), maximum (Max.), and references (refs.). * ND = nondetectable.

## Data Availability

No new data were created or analyzed in this study.
